# Research on Low-Voltage Arc Fault Based on CNN–Transformer Parallel Neural Network with Threshold-Moving Optimization

**DOI:** 10.3390/s24206540

**Published:** 2024-10-10

**Authors:** Xin Ning, Tianli Ding, Hongwei Zhu

**Affiliations:** 1 State Grid Sichuan Electric Power Research Institute, Chengdu 610041, China; x.ning@stu.xjtu.edu.cn; 2 Power Internet of Things Key Laboratory of Sichuan Province, Chengdu 610041, China; 3 College of Integrated Circuits, Zhejiang University, Hangzhou 311200, China; zhuhw@zju.edu.cn

**Keywords:** arc fault, CNN, Transformer, parallel neural network, threshold moving

## Abstract

Low-voltage arc fault detection can effectively prevent fires, electric shocks, and other accidents, reducing potential risks to human life and property. The research on arc fault circuit interrupters (AFCIs) is of great significance for both safety in production scenarios and daily living disaster prevention. Considering the diverse characteristics of loads between the normal operational state and the arc fault condition, a parallel neural network structure is proposed for arc fault recognition, which is based on a convolutional neural network (CNN) and a Transformer. The network uses convolutional layers and Transformer encoders to process the low-frequency current and high-frequency components, respectively. Then, it uses Softmax classification to perform supervised learning on the concatenated features. The method combines the advantages of both networks and effectively reduces the required depth and computational complexity. The experimental results show that the accuracy of this method can reach 99.74%, and with the threshold-moving method, the erroneous judgment rate can be lower. These results indicate that the parallel neural network can definitely detect arc faults and also improve recognition efficiency due to its lean structure.

## 1. Introduction

In the power system, low-voltage arc faults are one of the potential safety hazards that can cause a series of accidents, such as equipment failures and home fires, resulting in hundreds of deaths and millions of cases of property damage. Therefore, to safeguard personal safety, prevent fire accidents, and maintain the normal operation of the power system, it is of great significance to detect arc faults and then trip the circuit breaker in time. The research on AFCIs and their algorithms helps to guide the high-efficiency identification of arc faults.

With the ever-increasing sorts of industrial equipment and household appliances, the current detection methods of arc faults can be affected by load characteristics. Household appliances can be roughly classified into three categories: the resistive category, the capacitive–inductive category, and the switching category. There are large differences in the current waveform and amplitude among these categories. Due to the complicated harmonic characteristics and stochasticity, it is difficult to distinguish arc faults from the normal load current, whether it is a series arc or a parallel arc. For example, in the 1~100 kHz frequency band, which is commonly used in arc detection, there is low-frequency interference and noise interference generated by switching load types, such as inverters, rectifiers, and DC/DC converters. The high-frequency component generated by arc faults is an important indicator of fault detection. However, for switching loads, their noise interferences, overlapped with the high-frequency component generated by the arc, affect the neural network’s identification, and thus, lead to a significant increase in the misjudgment rate.

The current identification approaches generally adopt machine learning methods, including support vector machines, neural networks, and other algorithms on the basis of extracting the time-frequency domain features of the current waveform. In [1], based on the multi-sensor method, Fourier transform is used to analyze the variety of loads. This method combines time-domain features, such as the current mean value, and frequency-domain features, such as harmonic factors, followed by training a convolutional neural network (CNN) with the preprocessed data. Nevertheless, its testing process is only for a single dimmer, where the generalization ability and the overall recognition ability are insufficient. Meanwhile, discrete wavelet transform (DWT) is used to provide a multiple-resolution analysis of the current signal in [2,3], which researches the influence of the decomposition degree, wavelet function, and other parameters on feature extraction. However, the number of load types used in training is quite small, and thus the method cannot fully meet the needs of various load types in practical applications. The authors of [4,5] converted the current waveform into two-dimensional images using short-time Fourier transform (STFT) and Gramian angular field (GAF), respectively. But STFT has the disadvantage of having a fixed window length. Also, the complexity of high-dimensional features in the GAF method requires a deep network to support training. In addition, a recurrent neural network (RNN) is employed to mine the potential timing information in the current. It is indicated in [6] that the CNNs and the long short-term memory (LSTM) network can be combined in series to explore the potential of the two methods. However, in this series method, it is difficult to divide the LSTM time steps in a meaningful way. Deep networks, such as the residual convolutional network (ResNet) [7], and ensemble learning methods, such as stacking [8], have also recently been applied to arc fault identification, where unextracted domain features can be directly used as network inputs. But in practical application, deep networks are easily limited by their computational complexity, and blindly increasing their depth leads to higher requirements for the computing power of embedded devices.

Notably, most research only focuses on how to improve the accuracy of arc fault detection. But the cost of misjudging the normal state as the fault state is much higher than that of not recognizing the fault. This unbalanced cost, named cost sensitivity, is not specifically described in most studies. Therefore, aiming to solve the noise problem above and reduce the misjudgment rate with small network complexity, an arc fault identification algorithm based on a parallel neural network is presented.

Following the idea in the above papers of combining multiple features, this method is expected to extract high-dimensional information through a CNN and a Transformer network and add threshold moving to flexibly adjust the model’s sensitivity to misjudged data. This dual-parallel structure takes the methods’ respective characteristics into consideration. For instance, the CNN has a strong feature extraction ability, high precision, and good training efficiency. And compared to an RNN, the Transformer can analyze time series features with parallel computation. This proposed parallel network feeds data into the CNN and Transformer encoders, independently abstracting arc fault characteristics from the perspective of image and time series. Finally, the outputs of the two are concatenated and classified by the fully connected layer and the Softmax layer to obtain the result.

## 2. Arc Fault Dataset and Detection Methods

### 2.1. Feature Extraction of Arc Current Waveforms

**Figure 1:**
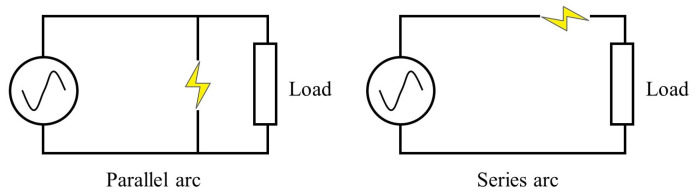
Types of arc faults.

Arc faults in our dataset can be divided into parallel and series arcs. The parallel arcs always occur between conductors with sudden current rising, while the current amplitude of the series arc will not increase significantly, which means that the series arcs have higher requirements in feature extraction. The two arcs are shown in Figure 1 below.

**Figure 2:**
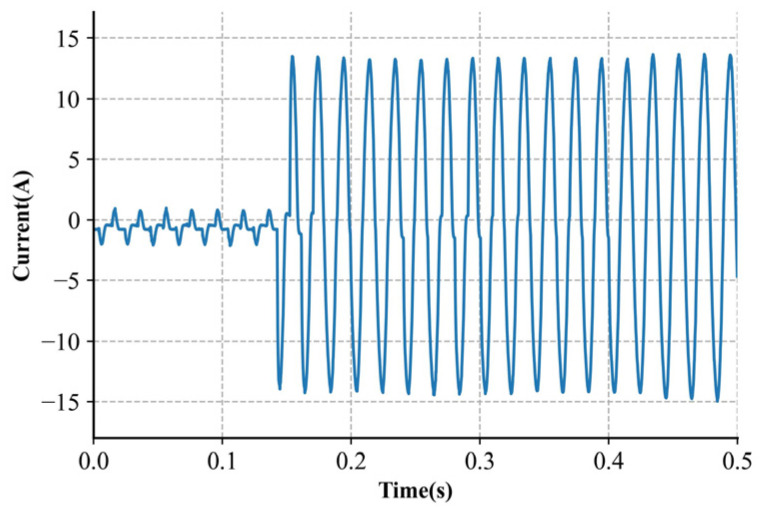
Appliance current waveforms with a parallel arc.

The detection of parallel arcs is a relatively simple task. The result of the parallel arcs can be seen as the bypass increase in Figure 1. Figure 2 illustrates the significant rise in current.

**Figure 3:**
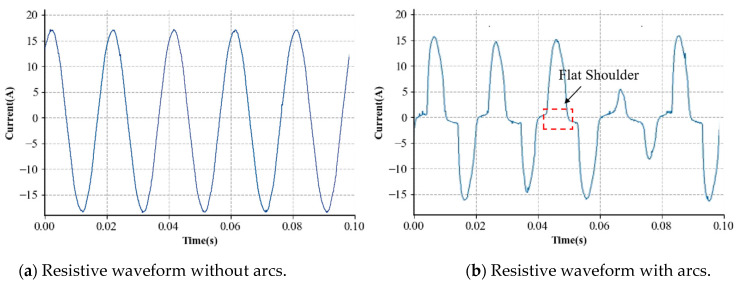

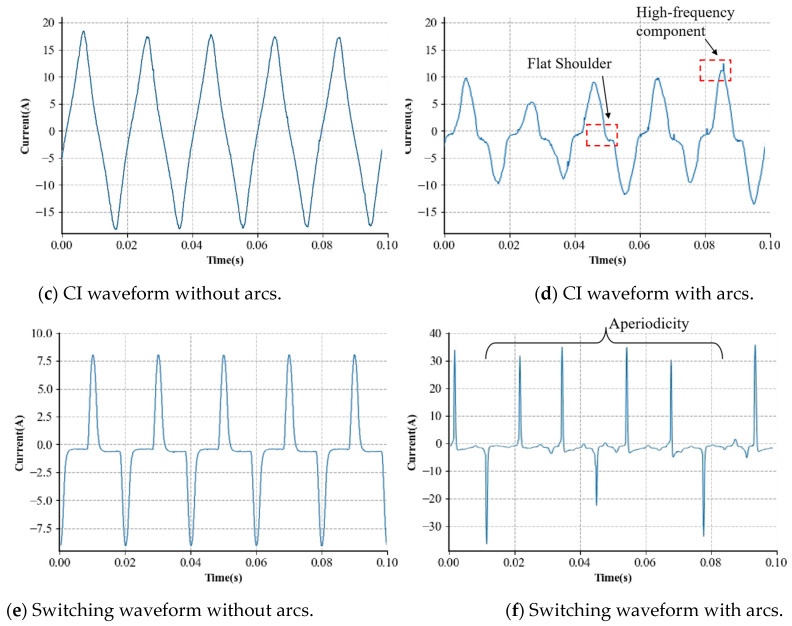
Appliance current waveforms with and without series arcs. (**a**,**b**) are from a resistor, (**c**,**d**) are from an electric drill, and (**e**,**f**) are from a switching mode power supply. To show the aperiodicity, (**f**) is displayed for a longer period.

For series arcs, loads are generally categorized into the following types: resistive, capacitive—inductive (CI), and switching loads [9]. In the existing research, the high-frequency features of current waveforms are typically used to determine the occurrence of arcs [10]. However, due to EMC interference, a high-frequency component may also appear during the normal operation of the switching devices. Relying solely on the high-frequency component for arc detection may lead to a false positive from confusion with a normal current. Therefore, processing low-frequency current waveforms, in particular, significantly enhances the recognition accuracy. This study examines eight typical loads across three categories: resistor, dimming lamp, switching mode power supply, halogen lamp, fluorescent lamp, electric drill, and vacuum cleaner, to gather current waveforms for both arc and non-arc conditions. The typical current waveform of these three types is illustrated in Figure 3.

Figure 3 shows that the arc fault current waveforms differ markedly from the standard current waveforms, exhibiting various characteristics depending on the load type. The usual operating current is a standard sine wave for purely resistive loads, such as resistors and halogen lamps. But, under arc situations, high-frequency spikes and flat shoulders may appear. High-frequency harmonics can, to some extent, indicate the presence of arcs. The flat shoulder feature also suggests small fluctuations in the current waveform near zero, indicating arc extinguishment and reignition around the voltage crossing zero point. Similar conclusions can be drawn for capacitive–inductive loads like drills. However, for switching devices, like switching mode power supplies and dimming lamps, the normal current already exhibits features like small slopes at zero point and noise compared to purely resistive loads. Arc detection relying solely on these features can result in substantial errors.

Another fundamental feature of arc faults is the variation between cycles, which demonstrates significant instability and varies noticeably across different load types. Particularly in switching devices during the arc faults occurring, the current’s amplitude and waveform show high irregularity. For instance, when an arc exists in a vacuum cleaner, the amplitude between cycles shows no stable pattern, with differing magnitudes in forward and reverse currents and prolonged zero-crossing times in some cycles.

Aperiodic low-frequency arc fault currents place specific demands on the generalization ability of the detection model: the model must not only distinguish aperiodic differences but also be compatible with various load types. The CNN fulfills the need to extract these abstract features. By stacking long-period signals into images, convolutional kernels can extract both intra-cycle features like flat shoulders and inter-cycle unstable features to achieve arc recognition. However, convolutional kernels are limited to extracting local features and lack long-term dependencies. Although the differences in adjacent cycle arcs may be minimal, arc phenomena can be observed in historical data. Therefore, the Transformer architecture is further introduced to process data in a time-series manner.

### 2.2. Fault Arc Acquisition and Dataset Division

**Figure 4:**
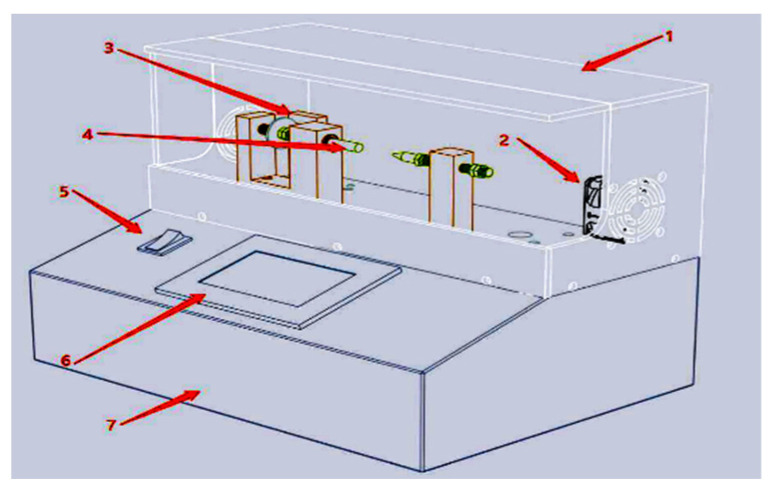
Structure of an arc generator. (1) Shield. (2) Exhaust fan. (3) Zero point sensor. (4) Tungsten copper electrode. (5) Main switch. (6) Human–computer interactive display. (7) Chassis.

Our dataset was obtained by collecting the current data from various loads in the laboratory, including the normal working state, series arc state, and parallel arc state. The arc generator, whose main structure is depicted in Figure 4 below, is experimental equipment to verify the actual arc characteristic combination and observe the arc burning phenomenon in the circuit. Upon connecting the arc generator and circuits in series or parallel, the measured data were obtained by ADC sampling. Four rows make up the data that the ADC sampled. The first row shows the low-frequency waveform of the current, and the second and the third rows obtained by filtering the high-speed sampling data indicate the current’s high-frequency characteristics. The last row labels whether a single half-cycle has an arc fault.

It is noteworthy that the arc faults cause the current data to be disturbed, and the fluctuation of the data between different half-cycles is one of the most critical factors in judging fault samples. Considering the effect of historical data and improving the algorithm accuracy, the sliding window method was adopted by combining historical half-cycle data with the current data. The sample duration in the constructed dataset was 0.5 s, which contains 50 half-cycle data, and the number of arcs determines the sample label. In an arc fault sample, we set the arc’s occurrence threshold to 8, meaning that if the threshold was exceeded, the sample was labeled as an arc fault. All samples with an arc were taken as positive samples, and the corresponding label was 1. The arc-free samples were regarded as negative examples, and their corresponding label was 0.

**Figure 5:**
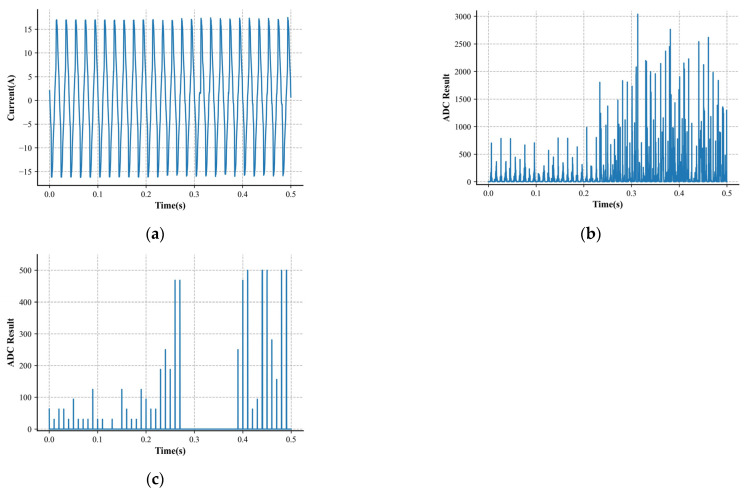
Characteristics of an arc sample. (**a**) Low-frequency current. (**b**) High-frequency characteristic 1. (**c**) High-frequency characteristic 2.

**Figure 6:**
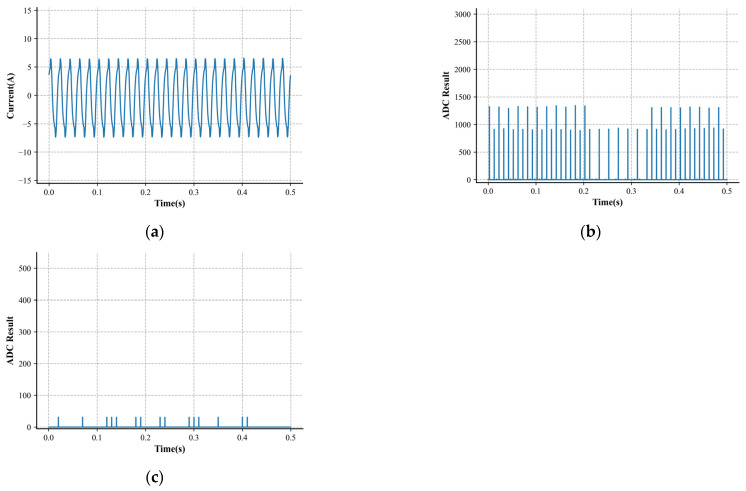
Characteristics of a normal sample. (**a**) Low-frequency current. (**b**) High-frequency characteristic 1. (**c**) High-frequency characteristic 2.

The above current data were randomly split into a train set, a validation set, and a test set at a ratio of 8:1:1. We needed the train set to fit the model and used the validation set to evaluate the model’s performance on previously unseen data and over-parameter adjustment. The test set predicted and reflected the model’s generalization ability in real scenarios after training. The constructed outcome of splitting is shown in Table 1. Figure 5 and Figure 6 show the specific samples of the load waveforms collected in the dataset.

### 2.3. Parallel Neural Network Structure

#### 2.3.1. Flow Chart of the Parallel Neural Network

Among the three features stated in Section 2.2, the low-frequency feature mainly depicts how the current waveform varied inside a half-cycle wave with an arc fault. It exhibited significant differences among the different load types and fault arcs, indicating a high level of complexity. In contrast, the high-frequency features captured in the latter two exhibited only brief fluctuations at a few time points, indicating a lower level of complexity. From a computer vision perspective, arc data can be reorganized into images, where each image row represents the data for a single cycle. Different channels represent low-frequency waveforms and high-frequency features extracted using convolutional kernels to capture features within cycles, between cycles, and across channels.

From a time-series perspective, the Transformer encoder was designed using a self-attention mechanism. Compared to convolutional local perception, self-attention has a shorter maximum path length, focusing on the global sequence and better handling data’s long-term dependencies.

**Figure 7:**
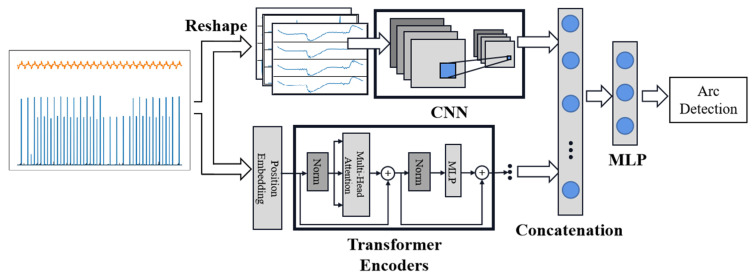
Flow chart of parallel neural network. Path 1 is designed based on a CNN, and Path 2 applies the Transformer encoders.

Based on the characteristics of the two network architectures, a dual-path parallel network structure is proposed, as illustrated in Figure 7. This structure can integrate high-dimensional spatial and temporal features to extract arc fault data comprehensively [11]. On the one hand, low-frequency current serves as an input for Path 1 after standardization. The original data are first reorganized into three channels, and each channel’s data are then reshaped into two-dimensional images. Multiple convolution and pooling layers are applied to train and learn complex parameters, capturing the potential features in the image domain. Simultaneously, the sequence is segmented into multiple time steps in Path 2, and each contains distinct feature vectors. After position embedding, these steps pass through the Transformer network to extract latent features over time. Following their separate extraction by the two paths, the load features are concatenated and then classified into two categories—with arc and without arc—through multiple fully connected layers and the Softmax layer, thereby realizing the complete algorithm flow for arc fault identification.

#### 2.3.2. Residual Block and Transformer Encoder

**Figure 8:**
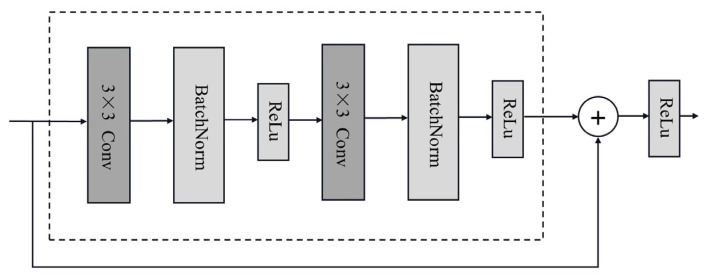
Structure of a residual block.

The structure of the CNN in our parallel neural network is based on the design of the ResNet [12], and its basic unit is a residual block, as illustrated in Figure 8. The core idea behind residual blocks is that the output of each block can achieve identity mapping, where the original input not only passes through these interconnected units for feature extraction but also directly adds to the output of the convolutional layers. This method allows the original input to propagate rapidly across layers. The residual connection method in the ResNet is widely used in computer vision and other fields. It addresses the vanishing gradient problem in deep networks and enhances the model’s expressivity and generalization. As a result, the model performs better than traditional convolutional networks, such as AlexNet [13].

The batch normalization (BatchNorm) layer in the residual block is added after the convolutional layer. The BatchNorm formula is given by Equation (1), as follows:

(1) $$\mathrm{BN}(x)=\gamma ⊙\frac{x-{\hat{\mu }}_{B}}{{\hat{\sigma }}_{B}}+\beta ,$$

where **x** represents a batch of input samples, ${\hat{\mu }}_{B}$ and ${\hat{\sigma }}_{B}$ stand for the batch sample mean and variance, and γ and β are the learnable scale and shift parameters with shapes consistent with the input. The BatchNorm serves as a regularization technique, standardizing the outputs of each layer in the network, accelerating the convergence speed, and further preventing overfitting. With the introduction of the residual structure, this path in the parallel network mainly consists of one convolutional layer, two pooling layers, and two residual blocks, ultimately outputting a 256-dimensional vector prepared for concatenation with the output of the Transformer encoder.

**Figure 9:**
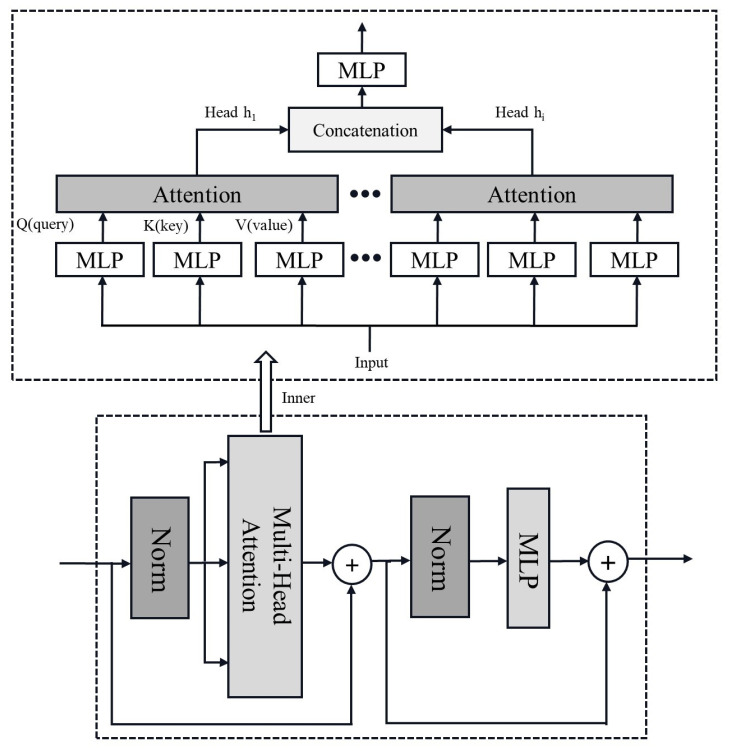
Structure of a Transformer encoder, which is the bottom half of the figure. The top half is the inner composition of the multi-head attention mechanism.

The Transformer structure is built upon the foundation of the attention mechanism, originally applied to text translation tasks to achieve sequence-to-sequence learning, and gradually expanded to fields such as language and computer vision [14]. For sequence-to-sequence tasks, both an encoder and a decoder are required, while for classification tasks, such as the judgment of arc faults in this paper, only the encoder portion is needed. The basic structure of the Transformer encoder is similar to that of the residual block. A structure resembling residual networks with residual connections is used within a single encoder. The key difference lies in its use of a self-attention mechanism to extract internal relationships in temporal signals. Additionally, the encoder block employs a regularization scheme named layer normalization rather than batch normalization. The structure of the encoder and its internal self-attention mechanism are shown in Figure 9.

In the computation of multi-head attention, the inputs serve as keys (**K**), values (**V**), and queries (**Q**) and then pass through fully connected layers to compute attention aggregation. When the evaluation function in the attention aggregation is specified as the Softmax function, the attention layer can be expressed as Equation (2):

(2) $$\mathrm{Attention}(Q,K,V)=\mathrm{softmax}(\frac{QK^{T}}{\sqrt{d_{k}}})V,$$

where the symbol *d_k_* represents the length of the keys, serving as the scale factor in the dot product. The operations above constitute the computation of a single attention head. In multi-head attention, each distinct attention computation is referred to as an attention head, and the number of attention heads is a hyperparameter that needs to be evaluated for its impact on training efficiency and computational performance. The multi-head attention mechanism feeds parallel input into the attention layer through several heads, then concatenates the output *h_i_* of the attention layer and passes it through a fully connected layer to obtain the final result. Unlike batch normalization, which normalizes within small batch samples, layer normalization operates on the feature dimensions using a similar formula. The difference in layer normalization is that the input **x** represents data on one feature dimension of an input sample, and the equation uses the mean and variance of this feature dimension.

### 2.4. Threshold-Moving Method

During the neural network training process, the weights of various sample classes are typically assumed to be equal. Nonetheless, in many practical applications, especially in the arc fault detection studied in this paper, the cost of misclassifying an arc’s presence or absence is unequal. This phenomenon is because detecting an arc fault in real-world scenarios requires multiple combined results. An occasional false negative has a relatively minor impact on the overall outcome; however, if a load misjudgment as a false positive leads to the circuit breaker cutting off, the impact on normal production and life far exceeds that of a false negative [15]. Cost-sensitive neural networks adjust sample weights through methods such as threshold moving, ensemble learning [16], MetaCost [17], and changing loss function [18] to reduce the consequences of classification errors.

In this experiment, the positive and negative ratio of data set samples reached about 1:4, which means that the training process learned more from the negative samples. The method of adjusting loss functions has minimal benefits. The threshold-moving method eliminates training process alterations and is a flexible solution for implementing cost sensitivity [19]. During the testing phase, for the Softmax outputs *O_i_* (i∈{0,1}) of a binary classification network, according to the definition of the cost-sensitive matrix, the threshold-moving method is carried out using Equation (3):

(3) $$O_{i}^{*}=\sum_{c=0}^{1}O_{i}\;\mathrm{Cos}t\;[i,\;c].$$

In Equation (3), $O_{i}^{*}$ represents the output after threshold moving, and Cost denotes the cost-sensitive matrix. Cost [*i*, *c*] represents the cost required to classify class *i* as class *c*. Since the objective of this problem is to reduce misjudgment costs, moving the threshold toward the lower-cost false negatives, Cost [0] = Cost [1] = 0, and Cost [0, 1] is fixed at 1. Thus, the above equation simplifies to Equation (4):

(4) $$\left\{\begin{array}{l} O_{0}^{*}=O_{0}\;\mathrm{Cos}t\;[0,\;1],\\ O_{1}^{*}=O_{1}. \end{array}\right.$$

Adjusting Cost [0, 1] can reduce the misjudgment rate, and its value needs to be precisely set based on the obtained model to ensure that both the false positive and false negative rates stay within acceptable ranges. The final classification results can be obtained by Equation (5):

(5) $$class=\underset{i}{\arg\max}O_{i}^{*}.$$

### 2.5. Training Scheme and Network Parameters

Following the method described in Section 2.3, the architecture of the constructed neural network is shown in Table 2, where all pooling operations are max pooling. The network’s output was classified using the Softmax layer, and the cross-entropy function calculated the loss on the training set. The optimizer was Adam, with a learning rate of 1.0 × 10^−4^, a weight decay parameter of λ = 1.0 × 10^−3^, and a batch size of 128. After training, the best-performing network on the test set was subjected to cost-sensitive optimization, determining the threshold-moving method’s parameters to achieve the model’s ideal misjudgment and false negative rate.

## 3. Experimental Results and Discussion

### 3.1. Training and Prediction Results

**Figure 10:**
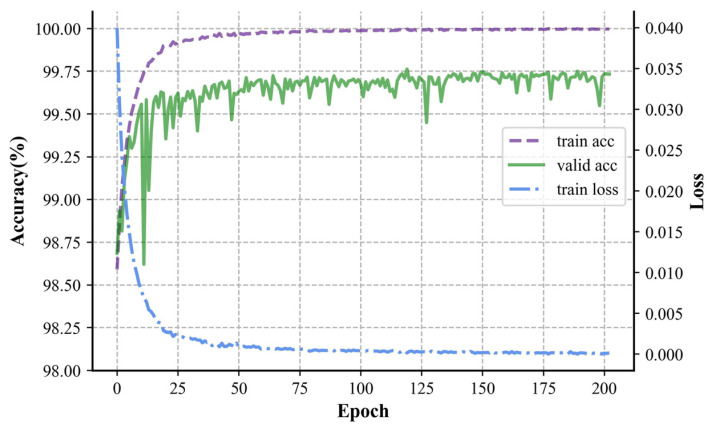
Plot of accuracy and loss with respect to epoch in training.

The dataset described in Section 2.2 was input into the network for training utilizing the hyperparameters outlined in Section 2.5. Randomization was applied to the data in the train set to avoid a fixed pattern in weight updating. The results of the training process with 200 iterations are illustrated in Figure 10.

During the initial stages of training, both the training and validation accuracies steadily rose, while the loss function steadily decreased. Due to measures like weight decay to suppress overfitting, the validation accuracy at this stage was higher than that of the test set. In the later stages of training, the training accuracy surpassed the validation accuracy, and after that, the training set continued to converge steadily, eventually reaching an accuracy of 99.9%. The upward trend of the validation set gradually flattened in the later stages, oscillating around 99.75%, and indicating that the train set was more fitted than the test set. From the standpoint of the training process, CNN’s high training efficiency and quick convergence and Transformer’s stability in training were absorbed by the parallel neural network, addressing issues like CNN’s oscillating accuracy and Transformer’s longer training times.

The optimum model with the highest accuracy on the validation set was picked as the best model for testing to mitigate the impact of overfitting. The trained classification model was then tested using the test set, and various metrics, such as accuracy, precision, recall, etc., were calculated from the confusion matrix to verify its feasibility for arc fault detection.

Given the presence of cost-sensitive issues, it was necessary to calculate the false positive rate (FPR) and false negative rate (FNR). FPR is defined as the proportion of non-arc data misclassified as arc data (false positive, FP), which is related to precision P and is calculated as 1-P; similarly, FNR is the proportion of arc data misclassified as non-arc data (false negative, FN), which is related to recall R and is calculated as 1-R. FPR and FNR were used as the metrics instead of precision and recall during the testing.

To better demonstrate our parallel network’s effectiveness, the two networks mentioned in the parallel network were trained independently, using the ResNet and the Transformer encoder separately for arc fault detection, and compared to our parallel network. Both models followed the same training scheme outlined in Section 2.5, and the ResNet added convolutional blocks and employed deeper layers. The performance differences in the test set after training all three models are shown in Table 3. The MACs column represents multiply–accumulate operations and indicates the algorithm complexity.

In Table 3, it can be observed that the parallel network model resolved the issue of the convergence difficulty encountered during the separate training of the Transformer encoder. Additionally, compared to the deeper ResNet, although both models had similar training set accuracy, the parallel network demonstrated advantages in the test set accuracy, false positive rate, and MACs. MACs represent the time consumption of the model when predicting arcs using CPU during forward propagation, clearly showing a notable difference in computational time between the parallel network and the ResNet. In practical applications, such as using models on microcontrollers to identify arc faults, CPU computation for deep networks can be time-consuming, presenting significant drawbacks regarding efficiency and real-time processing for arc detection. Our parallel network achieved higher accuracy with lower computational complexity, providing a neural network design approach for practical application scenarios.

To further illustrate the effectiveness of using the parallel neural network to handle high-frequency features and low-frequency current, the test results were compared with other papers, as shown in Table 4. The sample parameters generally influenced the identification accuracy of different methods in the dataset. However, in this study, even with a large dataset, diverse load types, and the inclusion of both series and parallel arc faults, our method still achieved high accuracy, demonstrating the superiority of incorporating high-frequency features and using a parallel neural network, as presented in our paper.

### 3.2. Cost-Sensitive Optimization

**Figure 11:**
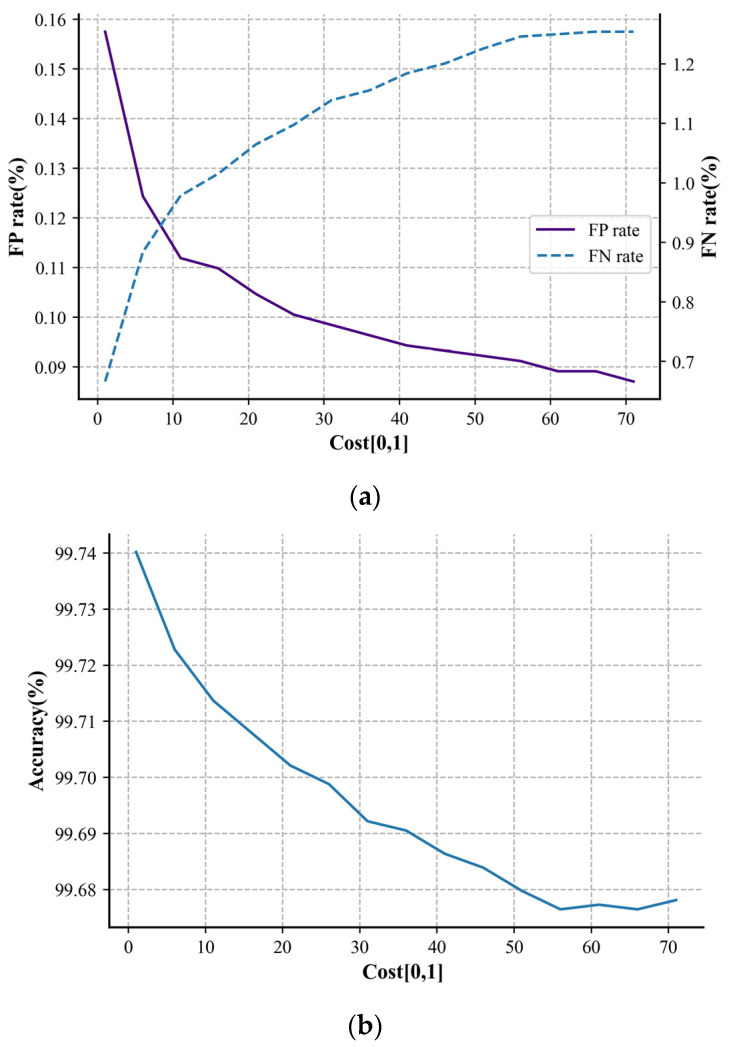
Plot of (**a**) FNR/ FPR and (**b**) accuracy with respect to the cost-sensitive parameter.

Using the threshold-moving method in Section 2.4, we transformed the identification of the test set into cost-sensitive optimization, further reducing the false positive rate of the parallel network model to better align with real-world scenarios. For selecting the appropriate parameters, Cost [0, 1] was initially set to 1 with a step size of 5, and 15 steps were applied to search for the model classification performance under different threshold-moving conditions. The results are shown in Figure 11.

The experimental results show that reducing the model’s false positive rate sacrificed the overall accuracy and the identification of the arc fault samples, but the effect was significant. When Cost [0, 1] was set to 60, the number of false positive samples on the test set decreased by nearly 45% compared to not using the threshold-moving method. In practical applications, based on the influence of cost-sensitive optimization on the results, selecting suitable cost-sensitive parameters can yield arc fault prediction results with lower false positive rates without changing the other network parameters or structures.

## 4. Conclusions

This paper proposes an arc fault detection method based on a parallel neural network combining a CNN and a Transformer. It processes low-frequency current waveforms while introducing high-frequency features and optimizes cost-sensitivity. The CNN extracts arc fault features from a visual perspective, while the Transformer encoder analyzes internal dependencies in time-series signals. Both networks independently extract abstract features and complete detection after concatenation, fully leveraging the respective advantages of the CNN and the Transformer.

Our experimental results demonstrate that the parallel network’s structure reduces computational complexity and performs competitively in prediction compared to other networks. The model’s overall accuracy on the test set reached 99.74% and exhibited excellent performance in the false positive rate. Moreover, it can flexibly adjust the false positive rate through the threshold-moving method, providing a neural network model design reference for the arc fault detection module in AFCIs.

Although the model in this study demonstrated excellent performance in terms of accuracy and FPR, some issues still need to be addressed. Firstly, the robustness of the model under extreme environmental conditions has not been sufficiently tested. Future improvements could enhance its adaptability to noise and enhancement techniques to increase its reliability in real-world applications. Additionally, incorporating more diverse datasets for training and validation would ensure the model’s generalization capability across different scenarios. Finally, further optimizing the model’s real-time processing ability, such as improving the complexity of the Softmax computation within the Transformer, will enhance its value in dynamic environments.

## Figures and Tables

**Table 1:** Dataset division and sample statistics.

Dataset	Normal Samples (Negative)	Arc Samples (Positive)	Total
Train set	769,654	195,279	964,933
Validation set	96,039	24,361	120,400
Test set	96,529	24,324	120,853

**Table 2:** Parameters of the parallel neural network.

Path	Layer Name	Output Size	Kernel Size
CNN	Input	3 × 25 × 128	—
	Conv1	64 × 13 × 64	64 × 7 × 7, stride 2
	Max pool	64 × 7 × 32	3 × 3, stride 2
	ResBlock 1	64 × 7 × 32	64 × 3 × 3
	ResBlock 2	256 × 4 × 16	256 × 3 × 3
	Average pool	256 × 1 × 1	4 × 16
	Flatten	256	—
Transformer	Position embedding	50 × 192	—
	4-Head encoders × 4	50 × 32	—
	Flatten + MLP	256	—
Path concatenation	Input	512	—
	Hidden layer 1	128	—
	Hidden layer 2	32	—
	Output	2	—

**Table 3:** Test and network identification results.

Model	Dataset	Accuracy (%)	FPR (%)	FNR (%)	MACs (M)
Transformer	Train set	99.80	0.1116	0.5623	0.21
	Test set	99.62	0.1865	1.1140	
ResNet	Train set	99.98	0.0008	0.0089	2.49
	Test set	99.68	0.1844	0.8633	
Parallel network	Train set	99.99	0.0001	0.0010	1.09
	Test set	99.74	0.1574	0.6660	

**Table 4:** Test accuracies of different methods.

	Parameter	Method				
		Our Method	Deep Residual Shrinkage Network with Attention [3]	Inception-V2 with SVM Loss Function [4]	CNN_LSTM [6]	1-D Dilated CNN [20]
Arc fault dataset	Sample frequency	6.4 kHz	25 kHz	10 kHz	50 kHz	50 kHz
	Dataset size	About 1 M	4240	1656	7200	About 100 k
	Preprocessing method	Extraction of high-frequency features	CWT + PCA	STFT	—	—
	Data dimensions	3 × 3.2 K	1 × 2.5 K	1 × 1 K	1 × 10 K	1 × 700
Model performance	Accuracy (%)	99.74	98.52	99.57	99.04	99.45
	FPR (%)	0.16	3.10	—	—	—
	FNR (%)	0.67	0.77	—	—	—

## Data Availability

The raw data used to support the findings of this study are available from the corresponding author upon request. The processed dataset and code are openly available at Kaggle at https://www.kaggle.com/datasets/tianliding2224/arc-fault-dataset (accessed on 1 October 2024).
